# Artificial Intelligence Algorithm-Based Magnetic Resonance Imaging to Evaluate the Effect of Radiation Synovectomy for Hemophilic Arthropathy

**DOI:** 10.1155/2022/5694163

**Published:** 2022-03-19

**Authors:** Heng Zhang, Shukai Duan, Wei Xiao, Xinyue Yang, Shenglin Li

**Affiliations:** ^1^College of Mathematics and Statistics, Southwest University, Chongqing 400715, China; ^2^College of Artificial Intelligence, Southwest University, Chongqing 400715, China; ^3^Department of Military Logistics, Army Logistic University of PLA, Chongqing 401331, China

## Abstract

This study aimed to discuss magnetic resonance imaging (MRI) based on artificial intelligence (AI) algorithm to evaluate the effect of radiation synovectomy for hemophilic arthropathy (HA). MRI based on the Canny algorithm was applied and compared with conventional MRI to evaluate its application effects according to the PSNR and SSIM. Sixty patients diagnosed with HA were selected as the research subjects. According to the detection method, the patients were divided into group A (pathological detection after radiation synovectomy), group B (conventional MRI detection), and group C (MRI detection based on the Canny algorithm). The application value of MRI based on the Canny algorithm was judged by comparing the differences between the two detection methods and pathological results. The results displayed that the reconstruction effect of the Canny algorithm was remarkably better than that of the traditional algorithm regarding the peak signal-to-noise ratio (PSNR) and structural similarity (SSIM), which showed a clearer synovial contour. The results of the IPSG score of joint effusion and hemorrhage showed that there was a difference in the detection rate of joints between conventional MRI and pathological results on the score of 1 and 2 (*P* < 0.05); and there was no significant difference between the MRI and pathological results based on the Canny algorithm (*P* > 0.05). The results of the IPSG score of synovial hyperplasia showed that the detection rate of conventional MRI was different from pathological results on the score of 1 and 2 (*P* < 0.05); and there was no significant difference between the MRI and pathological results based on the Canny algorithm (*P* > 0.05). The results of the IPSG score of hemosiderin deposition showed that the detection rate of conventional MRI was different from the pathological results on the score of 1 and 2 (*P* < 0.05); and there was no significant difference between the MRI and pathological results based on the Canny algorithm (*P* > 0.05). The synovial volume of patients after surgery was reduced compared with that before surgery. One-factor variance was used to analyze the clinical hemorrhage frequency before and after surgery, and the results showed that the differences were statistically significant (*P* < 0.05). Therefore, MRI on account of AI algorithm made it easier to detect synovial contour, which was helpful to evaluate the efficacy of polygenic risk scores (PRS) surgery in HA patients. MRI based on the Canny algorithm had less differences between the score of hemophilic arthropathy and pathological results, which could replace conventional MRI examination and have clinical application value.

## 1. Introduction

Hemophilia is an inherited blood clotting disorder. Hemophilic arthropathy (HA) first appeared in young children with repeated hemorrhage inside the joints, and blood accumulation triggered an inflammatory response in the joints. The accumulation of cytokines such as interleukin and tumor necrosis factor, and hemosiderin deposition eventually led to joint degeneration and damage, which was almost the common outcome of hemophiliacs [1, 2]. Deficiency of coagulation factors in hemophiliacs is caused by a deficiency of coagulation factor VIII (FVIII) (hemophilia A) or coagulation factor IX (hemophilia B) [3]. Because the prevalence of joint disease is the main factor that leads to joint deformity in hemophiliacs, many methods have been developed to determine the severity of HA. Clinical joint scores are usually used only for severe HA joints, but relatively light joint degeneration is not easily detected and is often seen in young patients [4]. 10%∼20% of children with severe hemophilia still have joint deformity, progressive arthritis, and multi-target joint involvement, which lead to reduced range of motion and joint mobility difficulties [5].

Early interventions are key to effective HA prevention. The major intervention is injecting coagulation factors into patients by timely preventive measures or on-demand treatment [6]. Repeated joint hemorrhage and long-term nonabsorption of hemophiliacs lead to synovitis or even permanent joint damage. Recurrent HA joint hemorrhage has been treated with surgery and synovectomy under arthroscopy in recent years. Both treatments are relatively expensive and require a large number of coagulation factor supplements. A long hospital stay and postoperative scarring can reduce joint mobility, which may have influence on further postoperative physical therapy [7]. Radiation synovectomy (RS) is one of the effective treatments for HA [8]. RS is performed mainly through introducing radioactive drugs into the articular cavity and destroying the sulfur synovial lining and pannus using radioactive *β*-ray and repairing the synovial lining to get it back to normal. RS can be performed on an outpatient basis without general anesthesia compared with surgery, and patients' medical costs are greatly reduced. Therefore, RS has become the main treatment for hemophiliac arthropathy with synovitis at present, and is mainly applicable to patients with hemophiliac arthropathy with synovitis [9].

At present, imaging detection has always been the main method for HA diagnosis [10]. In particular, magnetic resonance imaging (MRI) plays a very important role in the evaluation of HA. The scoring system of MRI is closely related to the evaluation of disease severity and clinical manifestations. MRI has high soft tissue resolution which can be used to detect synovial hyperplasia. Synovial inflammation can be enhanced by the intravenous injection of a gadolinium contrast agent during MRI. According to foreign reports, enhanced MRI is used to evaluate synovial hyperplasia volume in rheumatoid arthritis patients and whether it is active. Domestic scholars also have relevant studies about using enhanced MRI on early rheumatoid arthritis [11]. However, there are rare reports about the value of MRI on the evaluation of HA. In recent years, therapeutic modalities such as protecting the original joint or slowing joint degeneration have changed within the framework of an integrated nursing method. People pay more attention to the regular evaluation of the joint conditions of hemophiliacs. The aim was to detect early joint diseases, prevent clinically obvious arthropathy in children, and avoid or limit its progression in adolescents and adults. However, subclinical joint injury is rarely detected and identified because it is difficult to detect. With the popularization and development of computers, multimedia, and communication networks, digital image processing technology has gradually become a new industry. Applying image processing techniques to artificial intelligence can help reduce the sum of jobs for people. Among them, the Canny algorithm based on an artificial intelligence algorithm has been widely used because of its advantages of short operation time and relatively simple calculation process; and the detection results of the Canny algorithm are better than other edge detection algorithms.

This study was aimed to discuss AI algorithm-based MRI to evaluate the effects of radiation synovectomy for HA in order to better understand the progress and prognosis of joint lesions and provide strong imaging support for clinical treatment and rehabilitation.

## 2. Materials and Methods

### 2.1. Study Objects

Sixty patients with HA who were treated in the hospital from December 2017 to May 2020 were selected as the research subjects, aged 13–46 years old, with an average age of 22.57 ± 7.88 years. A total of 146 joints were included, including 84 knee joints, 38 ankle joints, 10 elbow joints, and 14 hip joints. The patients were randomly divided into group A (pathological detection after radiation synovectomy), group B (conventional MRI detection), and group C (MRI detection based on the Canny algorithm). All the patients in the study signed informed consents, and the study was approved by the ethics committee of the hospital.

Inclusion criteria were given as follows: first, the normal value of FVIIIC activity in normal people was 50%–150%, and it was confirmed by clinical and laboratory tests that the FVIIIC activity was less than 50% accompanied by the deficiency of FVIII or IX coagulation factor. Second, patients with hemophilia who have one or more target joints by clinical physical examination or X-ray examination are patients with hemophilic arthropathy, and those who had experienced more than one bleeding in the past 6 months. Third, the selected patients with hemophilic arthropathy were negative for HV (human immunodeficiency virus positive) and negative for hepatitis B virus surface antigen and hepatitis C antigen. The exclusion criteria were given as follows: first, patients were unable to undergo MRI detection. Second, patients had severe joint deformity. Third, the quality of patient image detection results was poor.

### 2.2. Detection Methods

MRI scans were performed using 3.0 T MRI machine. A knee coil was used for the knee joint, and an ankle coil was used for the ankle joint. Image T1-weighted image (T1WI) and proton density-weighted image-fat suppression (PDWI-FS) body coils were used for the elbow and hip joints, respectively. Both of them were 16-channel coils. Sagittal, coronal, and transverse scans were performed with turbo spin echo sequences (TI weight knee, elbow, and ankle joints), and coronal and transverse scans were performed for the hip joint. First, the time of repetition (TR) and the time of echo (TE) of T1WI of knee joint were 310 and 11 ms, respectively. The TR and TE of PDWI-FS were 4200 and 36 ms, respectively. The matrix was 384 × 384 and the deg field of view (FOV) was 16 cm × 16 cm. Second, the TR and TE of ankle *T*-weighted image (TWI) were 416 and 11 ms, respectively. The TR and TE of PDWI-FS were 4,180 and 33 ms, respectively. The matrix was 320 × 320 and the FOV was 15 cm × 15 cm. Third, the TR and TE of elbow T1WI were 498 and 10 ms, respectively. The TR and TE of PDWI-FS were 3000 and 22 ms, respectively. The matrix was 320 × 320 and the FOV was 15 cm × 15 cm. The TR and TE of the hip joint T1WI were 521 and 10 ms, respectively. The TR and TE of PDWI-FS were 3,000 and 50 ms, respectively. The matrix was 512 × 512 and the FOV was 38 cm × 38 cm. The layer thickness was 35 mm, the layer spacing was 0.4 mm, the nose-earlobe-xiphoid distance was 1, and the total scanning time of a single joint was about 10 min.

### 2.3. Detection Index and Grouping

According to the International Prophylaxis Study Group (IPSG) scoring criteria, MRI scores of joint effusion or hematocele, synovial hyperplasia, hemosiderin deposition, bone erosion, and bone cystic degeneration and cartilage damage were evaluated for all joints [12], which could be shown in Table 1.

### 2.4. Edge Detection Algorithm on account of AI

The edge detection algorithm was a basic topic for image understanding, analysis, and recognition. It provided the basis and the method for image segmentation, and had important study significance in the field of medical microscopic image understanding and pattern recognition. The Canny algorithm was used [13] because it consumed less time, and the process was relatively simple.

Gaussian filter smoothing image: *I*′=*I∗G*. *G*=*h*(*x*, *y*, *δ*) was a two-dimensional Gaussian function, and *I*′=*g*(*x*, *y*) was the smoothed image; *I*=*f*(*x*, *y*) was the original image and *∗* was a convolution.

The gradient amplitude and the direction of the image were calculated by the Gaussian first-order partial differential finite difference operator. Two convolution templates were used to carry out convolution operation on the image in the *χ* direction and the *γ* direction, respectively. The amplitude of the gradient could be obtained by calculating the sum of squares and the square root of the obtained convolution results. Finally, the gradient direction was obtained by an inverse trigonometric function operation.

A 2 × 2 first-order difference template was used to approximate the leading numbers *f*(*x*, *y*) of *χ* and *γ*, which are shown in the following equations:

(1) $$\begin{array}{c} f_{x}^{\prime }\left(x,y\right)=\left(-1\right)*f\left(x-1,y-1\right)+f\left(x,y-1\right)+1*f\left(x+1,y-1\right)+\\ \left(-2\right)*f\left(x-1,y\right)+0*f\left(x,y\right)+2*f\left(x+1,y\right)+\left(-1\right)*f\left(x-1,y+1\right)+0*f\left(x,y+1\right)+1*f\left(x-1,y+1\right)=\left[f\left(x+1,y-1\right)+2*f\left(x+1,y\right)+f\left(x+1,y+1\right)\right]\\ -\left[f\left(x-1,y-1\right)+2*f\left(x-1,y\right)+f\left(x-1,y+1\right)\right], \end{array}$$

(2) $$\begin{array}{c} f_{y}^{\prime }\left(x,y\right)=1*f\left(x-1,y-1\right)+2*f\left(x,y-1\right)+1*f\left(x+1,y-1\right)+0*\\ f\left(x-1,y\right)+0*f\left(x,y\right)+0*f\left(x+1,y\right)+\left(-1\right)*f\left(x-1,y+1\right)+\left(-2\right)*\\ f\left(x,y+1\right)+\left(-1\right)*f\left(x-1,y+1\right)=\left[f\left(x-1,y-1\right)+2*f\left(x,y-1\right)+f\left(x-1,y+1\right)\right]-\left[f\left(x-1,y+1\right)+2*f\left(x,y+1\right)+f\left(x+1,y+1\right)\right]. \end{array}$$

The mean of finite difference was detected in a difference template 2 × 2, so as to compute the partial gradient of *χ* and *γ* at the same point in the image. The gradient amplitude and azimuth are shown in the following equations:

(3) $$\begin{array}{c} \vartheta \left[x,y\right]=\sqrt{f_{x}^{\prime }{\left(x,y\right)}^{2}+f_{y}^{\prime }{\left(x,y\right)}^{2}}, \end{array}$$

(4) $$\begin{array}{c} \theta \left[x,y\right]=\text{arctan}\left(\frac{f_{x}^{\prime }\left(x,y\right)}{f_{y}^{\prime }\left(x,y\right)}\right). \end{array}$$

The gradient amplitude was suppressed by nonmaximum. A 3 × 3 window was on each central pixel. The center pixel of the field was compared with 2 pixels along the gradient direction, and was set to 0 if this point wasn't larger than the 2 adjacent points.

The connection edge was detected by the double threshold method. It was suggested to start with 2 definitions.

Def1: smoothness of function: a function with *n*-order differentiable and continuous *n*-order differentiable function was a smooth function.

Def2: approximation degree of function: the approximation degree of any 2 functions *f*(*x*),*g*(*x*) in the total square integrable function defined on [*a*, *b*] could be defined as

(5) $$\begin{array}{c} L=f-g_{2}={\left[\int_{a}^{b}{\left[f\left(x\right)-g\left(x\right)\right]}^{2}\right]}^{1/2}\text{d}x. \end{array}$$

*g*(*x*)=*f*(*x*)*∗G*(*x*), *g*(*x*), and *f*(*x*)*∗* represented the processed image, the original image, and the convolution, respectively. The differential property of convolution is shown in the following equation:

(6) $$\begin{array}{c} \frac{d^{n}g\left(x\right)}{dx^{n}}=\frac{d^{n}}{dx^{n}}\left[f\left(x\right)*G\left(x\right)\right]\\ =f\left(x\right)*\frac{d^{n}G\left(x\right)}{dx^{n}}. \end{array}$$

According to equation (6), another proposition was suggested: *a*(*x*)=*b*(*x*)*∗c*(*x*). *c*(*x*) was a smooth function of order ***n***, and it increased as *n* increased, which is shown in the following equation:

(7) $$\begin{array}{c} \vartheta =\int_{-\infty }^{+\infty }{\left|a\left(x\right)-b\left(x\right)\right|}^{2}. \end{array}$$

### 2.5. Image Quality Evaluation Criteria

At present, the common objective evaluation methods for reconstructed image quality include the peak signal-to-noise ratio (PSNR) and maximizing the structural similarity (SSIM) [14]. PSNR is the ratio of signal maximum power to signal noise power, which is generally used to evaluate the quality of superresolution reconstructed images. The following is the basic principle of PSNR: each pixel has a color value, which changes when the image is compressed and decompressed. PSNR could detect changes in color values, and signals have a wide dynamic range; thus, the PSNR value is expressed in decibels. The greater the PSNR value between the two images, the higher the similarity between the two images. For a clear image *I* and a noisy image *J* of the same size *m* × *n*, the mean square error (MSE) between the two graphs is defined as

(8) $$\begin{array}{c} \frac{1}{mn}\sum_{i=0}^{m-1}\sum_{j=0}^{n-1}{\left[I\left(i,j\right)-J\left(i,j\right)\right]}^{2}. \end{array}$$

In the above equation, *n* represents the binary number of the image pixel value, *m* represents the number of image samples in each training batch, and *i* and *j* are the indexes of *m* and *n*, which indicate a certain pixel point in the image. MSE represents the average sum of squares of the difference between the predicted and the true value. The definition of PSNR for a grayscale image could be obtained through MSE, which is shown in the following equation:

(9) $$\begin{array}{c} \text{PSNR}=10^{*}\log_{10}\left(\frac{{\text{MAX}}_{I}^{2}}{\text{MSE}}\right), \end{array}$$

where MAX1=2*B* − 1 is the maximum pixel value of an image in MSE. The smaller the MSE, the larger the PSNR and the better the image reconstruction quality.

SSIM is an indicator that measures the similarity between images before and after compression. Its calculation equations can be shown in the following equations:

(10) $$\begin{array}{c} \text{SSIM}\left(M,N\right)=l\left(M,N\right)\cdot c\left(M,N\right)\cdot s\left(M,N\right), \end{array}$$

(11) $$\begin{array}{c} l\left(M,N\right)=\frac{2\mu_{M}\mu_{N}+C_{1}}{\mu_{M}^{2}+\mu_{N}^{2}+C_{1}}, \end{array}$$

(12) $$\begin{array}{c} c\left(M,N\right)=\frac{2\sigma_{M}\sigma_{N}+C_{2}}{\sigma_{M}^{2}+\sigma_{N}^{2}+C_{2}}, \end{array}$$

(13) $$\begin{array}{c} s\left(M,N\right)=\frac{2\sigma_{MN}+C_{3}}{\sigma_{M}\sigma_{N}+C_{3}}. \end{array}$$

In equations (10)∼(13), *M* and *N* represent clear images and noisy images, respectively. *μ*_*M*_ and *μ*_*N*_ represent the average of *M* and *N,* respectively. *σ*_*M*_^2^ and *σ*_*N*_^2^ represent the variance of *M* and *N,* respectively. *σ*_*MN*_ represents the covariance of *M* and *N*. *Q*_1_, *Q*_2_, and *Q*_3_ represent the covariance of *M* and *N* to avoid dividing by zero. *L* is the range of the pixel value (225) in *Q*_1_=(*k*_1_ × *L*)^2^, *Q*_2_=(*k*_2_ × *L*)^2^, and *Q*_3_=*Q*_2_/2. *K*_1_=0.01 and *K*_2_=0.03 are the default values.

The specific calculation of *μ*_*M*_, *σ*_*M*_^2^, and *σ*_*MN*_ is shown in the following equations:

(14) $$\begin{array}{c} \mu_{M}=\frac{1}{H\times W}\sum_{i=1}^{H}\sum_{j=1}^{W}M\left(i,j\right), \end{array}$$

(15) $$\begin{array}{c} \sigma_{M}^{2}=\frac{1}{H\times W-1}\sum_{i=1}^{H}\sum_{j=1}^{W}{\left(M\left(i,j\right)-\mu_{M}\right)}^{2}, \end{array}$$

(16) $$\begin{array}{c} \sigma_{MN}=\frac{1}{H\times W-1}\sum_{i=1}^{H}\sum_{j=1}^{W}{\left(M\left(i,j\right)-\mu_{M}\right)}^{2}. \end{array}$$

In the calculation, a *H* × *H* window was given; it was suggested to slide the window on the image continuously for calculation. Finally, the average value was taken as the overall SSIM value.

### 2.6. Statistical Methods

SPSS22.0 statistical analysis software was used for data processing, and mean ± standard deviation was used for measurement data. The *T* test was used for data satisfying normal distribution. The mean synovial volume before and after surgery was compared; the paired *t*-test of two samples was used, and *P* < 0.05 indicated the differences were statistically significant. Counting data were compared by one-factor variance analysis, and *P* < 0.05 indicated the differences were statistically significant.

## 3. Results

### 3.1. Evaluation Results of Image Reconstruction

**Figure 1:**
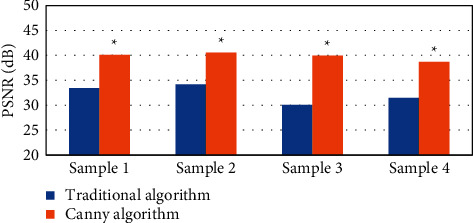
Comparison of the PSNR values between the two images (*∗* indicates that compared with conventional MRI processing, the difference was statistically significant (*P* < 0.05)).

**Figure 2:**
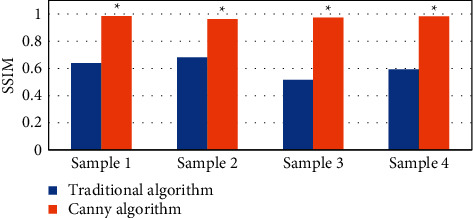
Comparison of the SSIM values between the two images (*∗* indicates that compared with conventional MRI processing, the difference was statistically significant (*P* < 0.05)).

**Figure 3:**
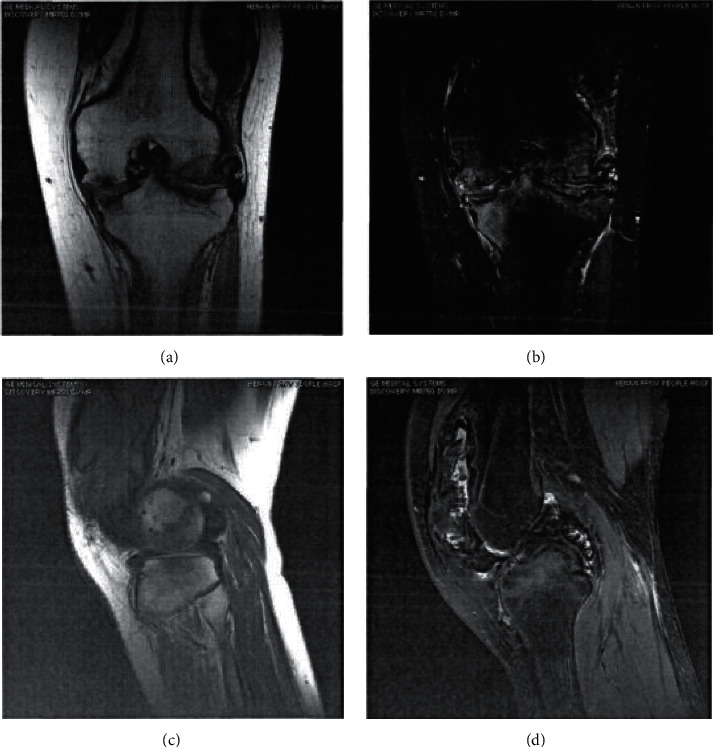
Comparison of scanning images of the two groups of HA patients showing synovium. (a, c) The images from an MRI detection. (b, d) The images from an MRI detection on account of the Canny algorithm.

The results showed that the Canny algorithm was significantly better than the traditional algorithm in PSNR and SSIM. Its reconstruction effect was remarkable, and the PSNR value and the SSIM index were higher. MRI on account of the Canny algorithm showed a more clear synovial contour, which was easy to detect and better to display, and could be shown in Figures 1–3.

### 3.2. Analysis of the IPSG Scoring Results by Different Methods of Joint Effusion and Blood

**Figure 4:**
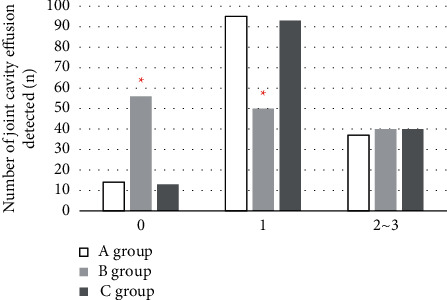
Results of joint effusion or hemoperfusion detection (*∗* indicates that compared with conventional MRI processing, the difference was statistically significant (*P* < 0.05)).

Postoperative, pathologically confirmed joint effusion and hemorrhage was scored as 0 for 14 joints, 1 for 59 joints, and 2∼3 for 37 joints. There was a difference in the detection rate of joints between conventional MRI and pathological results on the score of 1 and 2 (*P* < 0.05). There was no significant difference between MRI based on the Canny algorithm and the pathological results (*P* > 0.05), as shown in Figure 4.

### 3.3. Analysis of Synovial Hyperplasia and Hemosiderin Deposition IPSG Score by Different Methods

**Figure 5:**
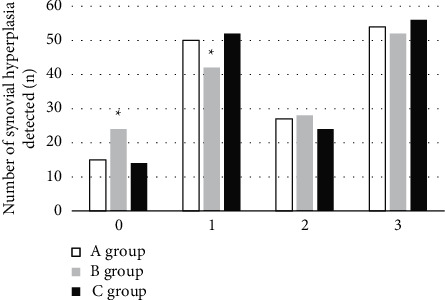
The number of detected joints in the two groups of synovial hyperplasia (*∗* indicates that compared with conventional MRI processing, the difference was statistically significant (*P* < 0.05)).

**Figure 6:**
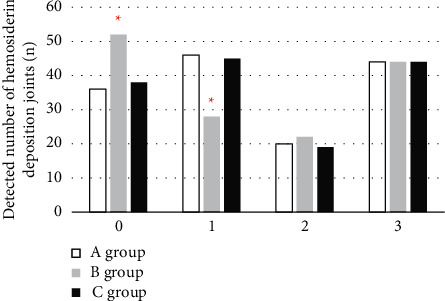
Hemosiderin deposition joint results of the two groups (*∗* indicates that compared with group A, the difference was statistically significant (*P* < 0.05)).

Postoperative pathological examination of synovial hyperplasia IPSG scores (Figure 5) showed that 15 joints were scored 0, 50 joints were scored 1, 27 joints were scored 2, and 54 joints were scored 3. There was a difference in the detection rate of joints between conventional MRI and the pathological results on the score of 1 and 2 (*P* < 0.05), and there was no significant difference between the MRI and pathological results based on the Canny algorithm (*P* > 0.05). Postoperative pathological detection of hemosiderin deposition IPSG scores (Figure 6) showed that 36 joints were scored as 0, 46 were scored as 1, 22 were scored as 2, and 44 were scored as 3. There was a difference in the detection rate of joints between conventional MRI and the pathological results on the score of 1 and 2 (*P* < 0.05); and there was no significant difference between the MRI and pathological results based on the Canny algorithm (*P* > 0.05).

### 3.4. Analysis of the Scoring Results of Bone Erosion and Cystic Degeneration IPSG by Different Methods

**Figure 7:**
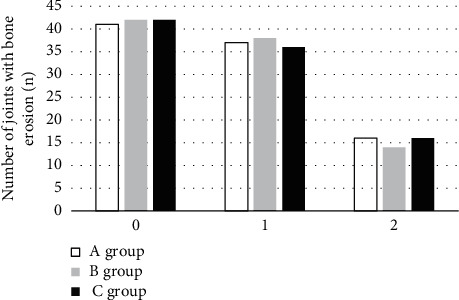
Bone erosion joint detection results in the two groups.

**Figure 8:**
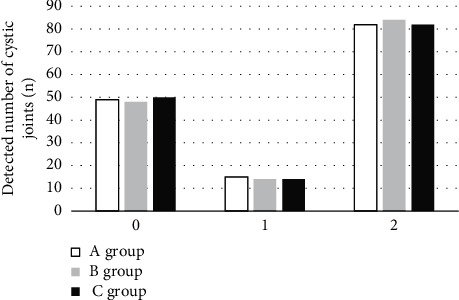
Bone degeneration detection results in the two groups.

In the IPSG score of “1”, “2”, and “3”, there was no statistical difference between the two groups in the detection results of bone erosion and bone cyst degeneration compared with the pathological results (*P* > 0.05). The specific results are illustrated in Figures 7 and 8.

### 3.5. Comparison of Synovial Volume Value before and after HA Surgery

**Figure 9:**
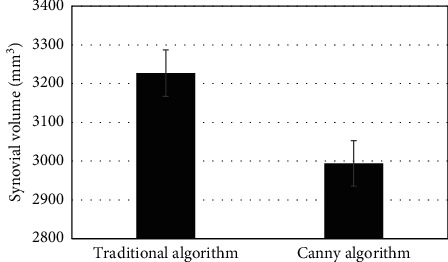
Comparison of synovial volume between plain scan group and enhanced group in the same patient.

The mean value of synovial volume obtained by the 2 treatments was compared, and the measured result was *P* > 0.05, indicating no statistical significance, which could be shown in Figure 9.

**Figure 10:**
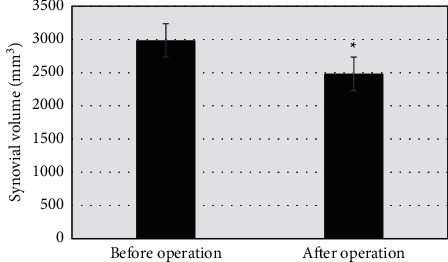
Comparison of synovial quantity before and after surgery (*∗* indicates that compared with preoperative results, the difference was statistically significant (*P* < 0.05)).

**Figure 11:**
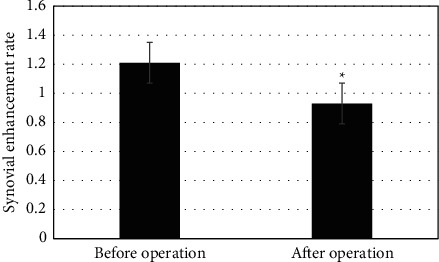
Comparison of synovial MRI enhancement rates before and after surgery (*∗* indicates that compared with preoperative results, the difference was statistically significant (*P* < 0.05)).

A total of 42 joints of 32 HA patients underwent PRS RS, and 28 of them were clinically effective. Joint swelling and pain disappeared or significantly alleviated, and hemorrhage frequency decreased. The other 4 patients underwent insignificant changes from the preoperative clinical symptoms. The comparison of synovial volume before and after surgery showed *P* < 0.05, which indicated statistically significant differences. After surgery, the extent of synovial enhancement in HA patients decreased compared with that before surgery; *P* < 0.05 indicated statistically significant differences. The results showed that synovial enhancement decreased significantly after surgery, which could be seen in Figures 10 and 11.

## 4. Discussion

Hemophilia is a hemorrhagic disorder with a recessive inheritance of sex chromosomes. Repeated joint hemorrhage leads to chronic hemophiliac arthritis and disability due to joint deformity, which is almost a common outcome for hemophiliacs [15]. The diagnosis and treatment of hemophiliacs are becoming more and more important as human beings have higher requirements for standard of living and quality of life. With the development and use of imaging equipment and new technology, imaging detection plays an increasingly important role in the diagnosis of HA. MRI has a significant advantage in evaluating the therapeutic effects of HA compared with traditional X-ray plain film. It can provide evidence of HA at an early stage or the occurrence of small structural degeneration. MRI score has also become a good, new method to monitor subtle HA lesions by imaging technology [16, 17] MRI not only has high tissue resolution, but also can detect synovial hyperplasia and hemosiderin deposition in pathological joints, and can clearly show the early abnormal degeneration of cartilage [18]. In this work, MRI based on the Canny algorithm was used to compare it with conventional MRI. The detection results showed that the reconstruction effect was remarkable, and a high PSNR value and SSIM index were obtained. The MRI image based on the Canny algorithm showed the outline of the synovium more clearly, which was easy to observe and better to display.

This study and the MRI IPSG scoring system made comprehensive assessments and quantification of arthropathy in terms of articular soft tissue and osteochondral lesions [19]. Articular soft tissue lesions included hemosiderin deposition, synovial hyperplasia, and joint effusion or hematocele. Osteochondral lesions included repeated joint articular cartilage injury, bone cystic degeneration, and bone erosion. The results showed that the IPSG score of joint effusion and hemorrhage showed that there was a difference in the detection rate of joints between conventional MRI and pathological results on the score of 1 and 2 (*P* < 0.05);there was no great difference between the MRI and pathological results based on the Canny algorithm (*P* > 0.05). The results of the IPSG score of synovial hyperplasia showed that the detection rate of conventional MRI was different from the pathological results on the score of 1 and 2 (*P* < 0.05);there was no significant difference between the MRI and pathological results based on the Canny algorithm (*P* > 0.05). The results of the hemosiderin deposition IPSG score showed that the detection rate of conventional MRI was different from the pathological results on the score of 1 and 2 (*P* < 0.05);there was no significant difference between the MRI and pathological results based on the Canny algorithm (*P* > 0.05). The MRI detection results based on the Canny algorithm are significantly more accurate. Therefore, clinical MRI on account of AI algorithm could replace traditional MRI for detection. MRI has high soft tissue resolution, especially hemosiderin deposition with extremely low signal, which makes it easier to detect. The results of this study showed that the difference between the two detection methods was not large, which may be related to the small number of samples in this study. MRI scores on account of AI algorithm were higher than those of traditional MRI in the quantitative scores of bone erosion or cystic degeneration in this study. The detection rate of lesions in MRI account of AI algorithm was higher than that in traditional MRI. Although the T2WPDW1-FS sequence of MRI showed a high signal of the cystic degeneration, which was easy to be detected, MRI detection had a relatively thick layer, which made it easy to miss some small lesions. Therefore, MRI on account of AI algorithm could replace traditional MRI for the IPSG scoring in bone erosion or cystic degeneration of HA patients. The narrowing of joint space was not obvious when cartilage lesions were only confined to the cartilage layer as the narrowing of the joint space was closely related to the loss of the full-thickness cartilage [20]. At this time, conventional MRI will underestimate cartilage damage, which is consistent with the findings of this study.

## 5. Conclusion

Compared with conventional MRI detection results, MRI reconstruction based on the Canny algorithm had a significant effect, obtained a higher PSNR value and SSIM index, and displayed a clearer synovial outline, which was easy to observe. It was more similar to the pathological test results and has higher accuracy, especially in the detection of mild abnormalities, which was more helpful for the evaluation of the efficacy of PRS surgery in HA patients. However, the sample size of the studies included was small, and the accuracy of some test results may be biased to a certain extent, which needs to be further explored in subsequent studies.

## Figures and Tables

**Table 1:** IPSG MRI scoring system.

Lesions		Degree		Score	
Joint effusion or bleeding hemorrhage		Small		1	
		Medium		2	
		Large		3	
Synovial hyperplasia		Small		1	
		Medium		2	
		Large		3	
Hemosiderin deposition		Small		1	
		Medium		2	
		Large		3	
Erosion of the subchondral cortex or edge of the joint surface		Any surface erosion		1	
		Bone erosion of at least 50% of a single bone		1	
Subchondral bone cystic degeneration		A single capsule		1	
		Coverage of more than 2 bones or more than 1/3 of a single bone		1	
Cartilage damage		Damage of cartilage anywhere		1	
		Loss of at least 50% cartilage volume in a single bone		1	
		Focal full-thickness cartilage loss of at least 1 bone		1	
		Loss of full-thickness cartilage covering at least 50% of a single bone		1	
Total				17 (9/8)	

## Data Availability

The data used to support the findings of this study are available from the corresponding author upon request.
